# SCREENING FOR NEUROPSYCHOMOTOR AND SOCIAL-EMOTIONAL DEVELOPMENT IN CHILDREN UNDER 24 MONTHS OF AGE IN THE BRAZILIAN SEMI-ARID REGION

**DOI:** 10.1590/1984-0462/2022/40/2020172

**Published:** 2021-05-26

**Authors:** Artemizia Francisca de Sousa, Maísa de Lima Claro, Patrícia Helen Carvalho Rondó

**Affiliations:** a Universidade Federal do Piauí, Campus Senador Helvídio Nunes de Barros, Picos, PI, Brazil.; b School of Public Health, Universidade de São Paulo, São Paulo, SP, Brazil.

**Keywords:** Child development, Child behavior, Primary health care, Desenvolvimento infantil, Comportamento infantil, Atenção primária à saúde

## Abstract

**Objective:**

To screen children under 24 months of age for neuropsychomotor and social-emotional development in a municipality of the Brazilian semi-arid region, using the Survey of Well-Being of Young Children (SWYC) scale.

**Methods:**

This is a quantitative cross-sectional study with a non-probabilistic sample, involving children aged 1 to 24 months and their respective mothers, recruited from primary care services in the municipality of Picos, Piauí, Northeastern Brazil. The screening for neuropsychomotor and social-emotional development using the SWYC scale also provided information about the family context. In addition, we administered a questionnaire to assess the children’s demographic and socioeconomic factors. Descriptive data analysis was performed.

**Results:**

The sample consisted mostly of adult mothers (84.0%), with more than 8 years of schooling (83.3%), belonging to the C, D, and E socioeconomic classes (75.3%). The prevalence of suspected cases of delayed neuropsychomotor development and social-emotional changes was 12.7 and 42.2%, respectively.

**Conclusions:**

The results point to the existence of children at risk of delayed development, particularly in the social-emotional domain, reaffirming the need to adopt child development screening as a health service routine, with the implementation of appropriate intervention programs.

## RESUMO

**Objetivo:**

Realizar a triagem para desenvolvimento neuropsicomotor e socioemocional em crianças menores de 24 meses, em município da região do semiárido brasileiro, utilizando a escala *Survey of Wellbeing of Young Children* (SWYC).

**Métodos:**

Estudo transversal, com abordagem quantitativa, amostra não proba­bilística, envolvendo crianças entre 1 e 24 meses e suas respectivas mães, selecionadas em serviços de saúde da atenção básica do município de Picos, Piauí. A triagem do desenvolvimento neuropsicomotor e socioemocional com uso da escala SWYC também forneceu informações sobre o contexto familiar. Além disso, utilizou-se questionário para avaliar fatores demográficos e socioeconômicos das crianças. Realizou-se a análise descritiva dos dados.

**Resultados:**

A amostra foi constituída de mães em sua maioria adultas (84,0%), com mais de oito anos de estudo (83,3%), pertencentes às classes socioeconômicas C, D e E (75,3%). A prevalência de casos suspeitos de atraso no desenvolvimento neuropsicomotor e de alterações socioemocionais foi de 12,7 e 42,2%, respectivamente.

**Conclusões:**

Os resultados apontam a existência de crianças em risco de desenvolvimento, sobretudo socioemocional, reafirmando a necessidade da adoção da triagem para desenvolvimento infantil como rotina nos serviços de saúde, com a implantação de programas de intervenção apropriados.

## INTRODUCTION

Current estimates indicate that 43% of children under 5 years living in middle- and low-income countries are at risk of not reaching their full physical, cognitive, psychological, and/or social-emotional potential,^1^ compromising the human capital in a generational cycle that perpetuates social inequality.^2^ Reversing this scenario would result in rates of return of 7-10% throughout life, by means of better economic, education, health, and social indicators, in addition to a reduction in crime.^3^

Child development is a complex and sequenced process, resulting from genetic, biological, and environmental interactions. Many factors are considered potential risks for neurodevelopment, as they represent adversities that affect families and the broader socioeconomic context, jeopardizing health, nutrition, safety and protection, responsive care, and early learning. Among pre-natal risk factors, intrauterine growth restriction, lead exposure, maternal human immunodeficiency virus (HIV), and iodine and iron deficiencies stand out. In the postnatal period, they include: stunting, malaria, as well as factors that limit learning opportunities and early stimulation for the child, such as maternal depression, domestic violence, and institutionalization.^1^ ^,^ ^4^ ^,^ ^5^

Despite the relevance of the theme and its scientific and political advances, the implementation and record of screening for aspects interrelated to child development (motor function, cognitive ability, social-emotional development) are still scarce in health services due to obstacles ranging from time availability and qualified professionals to the lack of space and appropriate materials.^6^

This scenario could change if health services adopted comprehensive screening instruments with good psychometric properties. These instruments allow identifying suspected delays in child development in more than 70% of cases.^7^ In this regard, an option that has proven to be feasible and that has been recommended recently for adapting to the needs of primary care, in addition to addressing risk factors related to the family context, is the Survey of Well-Being of Young Children (SWYC) scale.^8^ ^,^ ^9^

This instrument screens for delays in neuropsychomotor development, behavioral changes, and family risk factors. It is available on-line for free, easy to use, completed in a short time, and does not require kits and specific training.^8^ Besides, it has national cut-off points to classify changes in neuropsychomotor and social-emotional development, seeking to meet the Brazilian sociocultural specificities.^9^

Monitoring child development in different populations in the first years of life, using feasible instruments, is a public health concern, necessary to support new policies. In addition, positive screening enables diagnostic assessments and the adoption of appropriate interventions. Given the above considerations, this study aimed to screen for the neuropsychomotor and social-emotional development of children under 24 months, using the SWYC scale, in a municipality of the Brazilian semi-arid, an original study in this region.

## METHOD

To prioritize the scientific rigor of the research and present clear, coherent, and concise findings, we adopted, from the study design planning to the writing of articles, the guidelines proposed by the Strengthening the Reporting of Observational Studies in Epidemiology (STROBE) initiative,^10^ which intends to improve the presentation of results of observational studies in epidemiology.

This cross-sectional study involved children aged 1 to 24 months and their respective mothers, recruited by non-probabilistic sampling from primary care services in the municipality of Picos, Piauí. The sample size was calculated based on the formula for cross-sectional studies with finite population,^11^ with a maximum sampling error of 5% and a 95% confidence level. We obtained a sample of 261 children under 2 years of age. The sample had a 10% increment to compensate for potential sample losses, resulting in a final number of 287 children.

The mother-child dyad was selected when they visited the services for routine appointments, vaccination, or pediatric care. Data collection was carried out between September and November 2019, by means of interviews conducted by trained Master’s and undergraduate students. The interviews took place preferentially in private rooms provided by the health services. If the participant had time constraints, a home collection was scheduled. All mothers who agreed to participate in the study or their guardians (in the case of mothers under 18 years) signed the informed consent form.

The inclusion criteria adopted were: children from the age group 1 to 24 months enrolled in primary care. The exclusion criteria were: having congenital malformations and/or genetic syndromes, evident neurological and/or sensory deficits, diagnosed musculoskeletal disorders, severe or debilitating chronic diseases, adoptive children, and those not accompanied by their mothers.

The present study was submitted to plataforma Brasil (a national database of research involving human beings) for evaluation by the Research Ethics Committee of the School of Public Health at Universidade de São Paulo, following legal and ethical recommendations for research involving human beings, obtaining the approval opinion number 3,502,243.

After the volunteers agreed and signed the relevant forms, they were asked to answer socioeconomic and demographic questions (maternal age, ethnicity, marital status - having a partner or not, schooling, paid work, per capita income, and socioeconomic class, according to the Brazilian Association of Research Companies - *Associação Brasileira de Empresas de Pesquisa*,^11^ as well as the child’s gender and age) and the SWYC scale, whose questions involve screening for neuropsychomotor development (developmental milestones), social-emotional development (baby pediatric symptom checklist - BPSC, and preschool pediatric symptom checklist - PPSC), and risk factors in the family environment.

The developmental milestones checklist consisted of 10 questions about motor, language, social, and cognitive development, with three possible and mutually excluding answers: not yet (0), somewhat (1), or very much (2). The classification involved comparing the total score (0 to 20 points) to a scoring guide. Data were categorized into yes or no for suspected delay in neuropsychomotor development (DNPMD). Children aged 1 to 3 months have no formal classification.

The BPSC, composed of 12 items, evaluated 3 domains: irritability, inflexibility, and difficulty with routines. Each question had three possible answers: not at all (0), somewhat (1), or very much (2). The cut-off point used was the 90^th^ percentile of scores obtained by Brazilian children, according to age and domain, corresponding to a total score close to 4 points.^9^

The PPSC comprised 18 questions (externalizing, internalizing, attention problems, and parenting challenges), with three possible answers: not at all (0), somewhat (1), or very much (2). The cut-off point used was the 90^th^ percentile of scores obtained by Brazilian children, according to age, with a total score close to 16 points corresponding to suspected changes.^9^

Thus, regarding the social-emotional development, the child demonstrated suspected changes when the screening was positive for any of the domains investigated in the BPSC (253 children aged 1 to 17 months) or the PPSC (34 children aged 18 to 24 months), with the overall prevalence presented for all children evaluated.

As to family risk factors, the mothers were questioned about: the use of cigarettes (one question - positive screening: “yes”), food insecurity (one question - positive screening: “sometimes true” or “often true”), suspected substance abuse disorder (three questions - positive screening: at least one “yes”), domestic violence (two questions - positive screening: more extreme option in at least one of the answers), reading for the child in the previous week, and maternal depression.

For the age group 1-9 months, maternal depression was assessed by the questionnaire “emotional changes with a new baby” (10 questions with 4 possible and mutually excluding answers, whose values ranged from 0 to 3 points. Positive screening: values greater than or equal to 10 points). For the remaining ages, two questions were asked with four possible and mutually excluding answers: “not at all” (0), “several days” (1), “more than half the days” (2), “nearly every day” (3), totaling 0 to 6 points. A total score greater than or equal to three points indicated positive screening.

We emphasize that this child development screening instrument was chosen for being the only one available suited for the logistical needs of the research performed. Fully validated instruments, with national cut-off points, and available in their entirety are still not accessible. Moreover, the SWYC scale can be used both in cross-sectional studies for screening and in longitudinal ones for developmental surveillance.^13^

Data were collected using the free platform Epicollect 5^®^ (Imperial College, London, UK), subsequently exported in .csv format, and organized in an Excel for Windows^®^ spreadsheet. This spreadsheet was imported into the Stata software, version 14.0 (Stata Corp., College Station, Texas, USA), followed by a variable consistency evaluation (identification of outliers and missing values), editing, and analysis.

A descriptive data analysis allowed us to characterize the participants, as well as present the prevalence rates of the outcomes investigated and their distribution, according to socioeconomic, demographic, and family environment aspects.

## RESULTS

**Figure 1:**
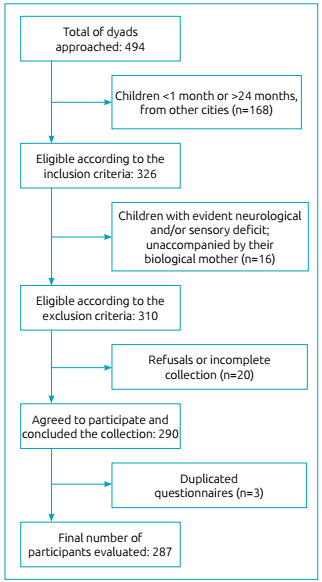
Flow chart of the number of mother-child dyads in the research.

A total of 494 mother-child dyads were approached in the 30 basic health units and the pediatric emergency department of the municipality. However, not all of them met the eligibility criteria, agreed to participate, or answered all research instruments, resulting in 287 participants. Thus, the losses recorded correspond to incomplete collections and duplicates (Figure 1). With respect to missing data, we found five missing responses for the income variable. In order to maximize the use of the information available, we performed a multiple imputation by predictive mean matching.

From a socioeconomic and demographic perspective, the sample proved to be homogeneous, with similar distribution between genders and prevalence of the age group 1-12 months. Concerning the family environment, except for the little reading done for children (22.6%), the prevalent conditions cited were favorable to child development (Table 1).

**Table 1:** Socioeconomic, demographic, and family environment characteristics (n=287).

		n	%
Maternal age	≤19 years	46	16.0
	>19 years	241	84.0
Ethnicity	White	69	24.0
	Non-white	218	76.0
Has a partner*	No Yes	35 252	12.2 87.8
Schooling	≤8 years	48	16.7
	>8 years	239	83.3
Paid work**	No Yes	213 74	74.2 25.8
Per capita income	≤0.5 MW	201	70.0
	>0.5 MW	86	30.0
Socioeconomic class	A and B C, D, and E	71 216	24.7 75.3
Child’s age (in months)	1-12	210	73.2
	12-24	77	26.8
Gender	Male	141	49.1
	Female	146	50.9
Exposure to cigarettes	No Yes	236 51	82.2 17.8
Alcohol and/or other illicit drugs	No Yes	275 12	95.8 4.2
Domestic violence	No Yes	243 44	84.7 15.3
Food insecurity	No Yes	236 51	82.2 17.8
Maternal depression	No Yes	252 35	87.8 12.2
Parents’ concerns	No Yes	111 176	38.7 61.3
Reads for the child	No Yes	222 65	77.4 22.6

*Civil or religious marriage, domestic partnership, or living together; **has paid work; MW: minimum wage.

The prevalence of suspected cases of DNPMD was 12.7%, with higher percentage differences in the child’s age variable - more frequent in the age group over 12 months (Table 2). Suspected change in social-emotional development was identified in 42.2% of the children assessed, with greater proportional differences among those exposed and not exposed to alcohol and/or illicit drugs (Table 3).

**Table 2:** Suspected delay in neuropsychomotor development according to socioeconomic, demographic, and family environment characteristics (n=213).

		No (n=186)	Yes (n=27)
		n (%)	n (%)
Maternal age	≤19 years	30 (78.9)	8 (21.1)
	>19 years	156 (89.1)	19 (10.9)
Ethnicity	White	45 (88.2)	6 (11.8)
	Non-white	141 (87.1)	21 (12.3)
Has a partner	No	24 (85.7)	4 (14.3)
	Yes	162 (87.6)	23 (12.4)
Schooling	≤8 years	26 (81.3)	6 (18.7)
	>8 years	160 (88.4)	21 (11.6)
Paid work	No	136 (86.1)	22 (13.9)
	Yes	50 (90.9)	5 (9.1)
Per capita income	≤0.5 MW	127 (86.4)	20 (13.6)
	>0.5 MW	59 (89.4)	7 (10.6)
Socioeconomic class	A and B	49 (89.1)	6 (10.9)
	C, D, and E	137 (86.7)	21 (13.3)
Child’s age (in months)	1-12	125 (91.9)	11 (8.1)
	12-24	61 (79.2)	16 (20.8)
Exposure to cigarettes	No	157 (88.2)	21 (11.8)
	Yes	29 (82.9)	6 (17.1)
Alcohol and/or drugs	No	178 (87.3)	26 (12.7)
	Yes	8 (88.9)	1 (11.1)
Domestic violence	No	175 (87.5)	25 (12.5)
	Yes	11 (84.6)	2 (15.4)
Food insecurity	No	151 (88.3)	20 (11.7)
	Yes	35 (83.3)	7 (16.7)
Maternal depression	No	167 (87.0)	25 (13.0)
	Yes	19 (90.5)	2 (9.5)
Parents’ concerns	No	72 (87.8)	10 (12.2)
	Yes	114 (87.0)	17 (13.0)
Reads for the child	No	135 (87.1)	20 (12.9)
	Yes	51 (87.9)	7 (12.1)

MW: minimum wage.

**Table 3:** Suspected change in social-emotional development according to socioeconomic, demographic, and family environment characteristics (n=287).

		No (n=166)	Yes (n=121)
		n (%)	n (%)
Maternal age	≤19 years	28 (60.9)	18 (39.1)
	>19 years	138 (57.3)	103 (42.7)
Ethnicity	White	40 (57.9)	29 (42.1)
	Non-white	126 (57.8)	92 (42.2)
Has a partner	No	17 (48.6)	18 (51.4)
	Yes	149 (59.1)	103 (40.9)
Schooling	≤8 years	26 (54.2)	22 (45.8)
	>8 years	140 (58.6)	99 (41.4)
Paid work	No	118 (55.4)	95 (44.6)
	Yes	48 (64.9)	26 (35.1)
Per capita income	≤0.5 MW	112 (55.7)	89 (44.3)
	>0.5 MW	54 (62.8)	32 (37.2)
Socioeconomic class	A and B	40 (56.3)	31 (43.7)
	C, D, and E	126 (58.3)	90 (41.7)
Child’s age (in months)	1-12	120 (57.1)	90 (42.9)
	12-24	46 (40.7)	31 (40.3)
Exposure to cigarettes	No	131 (55.5)	105 (44.5)
	Yes	35 (68.6)	16 (31.4)
Alcohol and/or drugs	No	157 (57.1)	118 (42.9)
	Yes	9 (75.0)	3 (25.0)
Domestic violence	No	142 (58.4)	101 (41.6)
	Yes	24 (54.5)	20 (45.5)
Food insecurity	No	141 (59.7)	95 (40.3)
	Yes	25 (49.0)	26 (51.0)
Maternal depression	No	148 (58.7)	104 (41.3)
	Yes	18 (51.4)	17 (48.6)
Parents’ concerns	No	66 (59.5)	45 (40.5)
	Yes	100 (56.8)	76 (43.2)
Reads for the child	No	127 (57.2)	95 (42.8)
	Yes	39 (60.0)	26 (40.0)

MW: minimum wage.

**Chart 1:**
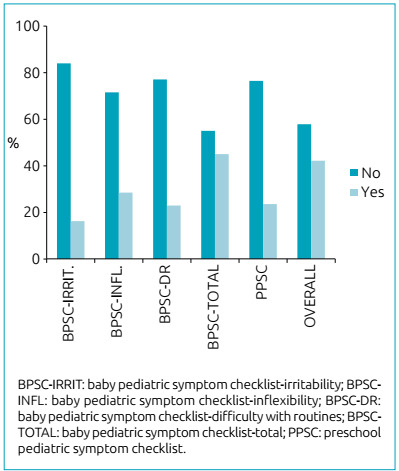
Suspected change in social-emotional development.

Among children under 18 months, 45% presented positive screening in at least 1 of the 3 aspects investigated. For those aged 18 months or older, 24% had difficulties regarding internalization and externalization, attention problems, and/or parenting challenges (Chart 1).

## DISCUSSION

The results indicate suspected DNPMD and especially behavioral changes in the children assessed, reinforcing the need for continuous surveillance so as to promote the timely adoption of interventions that optimize child development and allow the children to reach their potential.

Neuropsychomotor development represents the acquisition of motor, cognitive, language, and psychosocial skills, which have such a sequential order that delays may prevent the child’s progress. The importance of developmental surveillance has been increasingly evidenced.^4^ Nonetheless, we do not have population-based data on its overall national prevalence, only local-regional samples with different methodologies, precluding comparisons and the definition of a general overview on the issue in Brazil.

Besides, studies using the SWYC scale to determine the prevalence of suspected cases of DNPMD and social-emotional changes are scarce, particularly in the first two years of life. Usually, the available research does not use the full instrument and has many objectives, including the evaluation of psychometric properties with versions in different languages,^8^ ^,^ ^14^ ^,^ ^15^ the feasibility of its use in communities with specific characteristics (tribal communities),^16^ the comparison of its performance with other instruments^17^, and its incorporation into medical protocols.^18^

Thus, the prevalence of suspected cases of DNPMD (12.7%) could only be properly compared to that extracted from the thesis that determined the national cut-off points for the SWYC scale.^9^ Based on the results reported by the author, 45 (21.64%) of 208 children aged 4 to 24 months presented total scores lower than those considered suitable for the developmental milestones, values, therefore, higher than the ones found in the present study. However, the reason for this difference cannot be determined because the other variables present in both investigations that could be related to this outcome are described for all children (415 children aged 1 to 65 months and their guardians), not only for those under 24 months, preventing accurate comparisons.

Regarding the social-emotional development, which involves emotions, temperament, and confidence, built based on stimuli and responsive interactions, no publications were found in scientific journals of works carried out in the country using the SWYC scale and the national classification.

The data employed to compare the results originated from the thesis involving a normative study to determine the cut-off points^9^ and from a dissertation that evaluated the perceived maternal bond and the risk of changes in social-emotional development.^19^Nevertheless, they worked with different age groups. The first^9^ assessed 415 children aged 1-65 months in the South region of the country (Santa Catarina), while the second^19^ evaluated 221 children aged 1-36 months in Northeastern Brazil (Ceará). The prevalence of suspected cases of changes in social-emotional development among the children evaluated in this study (Chart 1) shows intermediate values between those found by Moreira^9^ (11.6, 12.1, 15.5, and 12.0% for irritability, inflexibility, difficulty with routines, and PPSC, respectively) and Rolim^19^ (33.0, 26.0, 37.0, 75.0, 40.0, and 51.0% for irritability, inflexibility, difficulty with routines, total BPSC, PPSC, and overall prevalence, respectively). North American publications using the SWYC scale report prevalence rates ranging from 28 to 47% for suspected cases of changes in social-emotional development.^20^ ^,^ ^21^ ^,^ ^22^

Other investigations conducted in Brazil presented varying degrees of prevalence of deficits in child development. Systematic review carried out in 2016, including publications from 2005 to 2015, found percentages of delayed development ranging from 0.0 to 46.3% in healthy children and from 14.2 to 100.0% in those with some pathological condition.^23^

This wide variation in prevalence rates may be partly explained by the screening instrument. The SWYC scale is administered to the caregiver who, despite the large knowledge of the theme and the unquestionable contributions to the evaluation process,^24^ can misinterpret the instrument due to personal perceptions - an aspect conditional on cultural determinants whose assessment is complex and was not the object of the present study - and inaccuracies related to recall and/or understanding bias.

The literature has evidence on the association between socioeconomic, demographic, and family factors and child development;^4^ however, these aspects were not evaluated in this study. We found the highest difference in prevalence of positive screening for DNPMD and social-emotional changes in the variables child’s age and exposure to alcohol and/or drugs, respectively.

The association between neuropsychomotor development and age was identified in other studies.^25^ ^,^ ^26^ ^,^ ^27^ Oliveira et al.^25^ and Silva et al.^26^ detected a greater likelihood of missed developmental milestones in children aged over 12 months. Older children have more experiences, which, if adverse, negatively influence the acquisition of skills expected for their age.

 Suspected cases of change in social-emotional development were more frequent among children not exposed to family environments with suspected substance abuse disorder, such as alcohol and/or drugs (42.9%), compared to those exposed (25.0%). This finding is unprecedented in the literature.

In addition, studies indicate that the caregiver’s health and disease status (depression, stress, anxiety, compulsive disorders, and other mental conditions, in addition to drug addiction), the lack of reading and learning moments with the child, family instability, marital conflict, domestic violence, and unfavorable socioeconomic and cultural conditions (adolescence, low schooling, and work availability) are risk factors for child development for exposing them to stressful, toxic situations, affecting the formation of affective bonds and/or jeopardizing the financial capacity of guardians to provide child care.^5^ ^,^ ^26^ ^,^ ^28^ ^,^ ^29^

Knowing and intervening in aspects related to child development is crucial, since they interfere with the quality of life and ability of social integration of the individual, both in childhood and adulthood, compromising the human capital transmitted to future generations and creating a cycle that can only be overcome by adopting intervention programs in early childhood to ensure that children have access to the means necessary to develop fully.^1^

The results of this investigation point to the existence of children at risk of delayed development, particularly in the social-emotional domain, reaffirming the need to adopt the screening as a health service routine to identify these cases in advance. The conclusions presented herein are limited to the children participating in this research. The sample originated from a semi-arid region of Northeastern Brazil, specifically the municipality of Picos, characterized by a high incidence of poverty; thus, the generalization of results requires replication in other regions.

The cut-off points used to determine the prevalence of suspected cases of changes in the neuropsychomotor and social-emotional domains were national values. However, they were defined in the South region of the country, in the municipality of Araranguá (Santa Catarina), whose human development index (HDI) (0.76), despite being similar to the Brazilian average (0.72), as pointed out by Moreira,^30^ is greater than that of Picos (0.69), site of the present study. This fact may represent a limitation, given that socioeconomic and cultural aspects can influence children’s performance in development tests. Possibly, only multicenter studies, in all Brazilian regions, will allow using cut-off points suitable to all Brazilian children.

## References

[1] Black MM, Walker SP, Fernald LC, Andersen CT, Digirolamo AM, Lu C (2017). Early childhood development coming of age: science through the life course. Lancet.

[2] Victora CG, Adair L, Fall C, Hallal PC, Martorell R, Richter L (2008). Maternal and child undernutrition: consequences for adult health and human capital. Lancet.

[3] Heckman J (2015). Four big benefits of investing in early childhood development. The Heckman Equation.

[4] Torquato IM, Dias HP, Lima AG, Faustino JK, Pontes FA, Maia MT (2019). Child development surveillance: analysis of risk factors for children under two years. Educ Ci Saúde.

[5] Sun J, Liu Y, Chen EE, Rao N, Liu H (2016). Factors related to parents’ engagement in cognitive and socio-emotional caregiving in developing countries: results from multiple indicator cluster survey 3. Early Child Res Q.

[6] Thomas RE, Spragins W, Mazloum G, Cronkhite M, Maru G (2016). Rates of detection of developmental problems at the 18-month well-baby visit by family physicians’ using four evidence-based screening tools compared to usual care: a randomized controlled trial. Child Care Health Dev.

[7] Glascoe FP (2015). Evidence-based early detection of developmental-behavioral problems in primary care: what to expect and how to do it. J Pediatr Health Care.

[8] Moreira RS, Magalhaes LC, Siqueira CM, Alves CR (2019). Cross-cultural adaptation of the child development surveillance instrument “Survey of Wellbeing of Young Children (SWYC)” in the Brazilian context. J Hum Growth Dev.

[9] Moreira RS (2016). Screening for developmental delay and behavioral changes: a normative study of the “Survey of Wellbeing of Young Children (SWYC)” in the Brazilian context.

[10] Elm E, Altman DG, Egger M, Pocock SJ, Gøtzsche PC, Vandenbroucke JP (2007). Strengthening the Reporting of Observational Studies in Epidemiology (STROBE) statement: guidelines for reporting observational studies. BMJ.

[11] Miot HA (2011). Sample size in clinical and experimental trials. J Vasc Bras.

[12] Associação Brasileira de Empresas de Pesquisa (2016). Critério de Classificação Econômica Brasil.

[13] Sheldrick RC, Perrin EC (2013). Evidence-based milestones for surveillance of cognitive, language, and motor development. Acad Pediatr.

[14] Gerdes M, Garcia-Espana JF, Webb D, Friedman K, Winston S, Culhane JH (2019). Psychometric properties of two developmental screening instruments for Hispanic children in the Philadelphia region. Acad Pediatr.

[15] Han D, Woo J, Jeong J, Hwang S, Chung US (2015). The Korean version of the pediatric symptom checklist: psychometric properties in Korean school-aged children. J Korean Med Sci.

[16] Whitesell N, Sarche M, Trucksess C (2015). The survey of well-being of young children: results of a feasibility study with American Indian and Alaska native communities. Infant Ment Health J.

[17] Oliver CR (2017). Getting the most out of pediatric screening. DNP Projects.

[18] Kells S (2018). Introducing screening for family risks in young children in primary care. Doctor of Nursing Practice. DNP Projects.

[19] Rolim LR (2018). The maternal bonding perception and the risk of changes in children’s socioemotional development / behavior.

[20] Iyer SN, Dawson MZ, Sawyer MI, Abdullah N, Saju L, Needlman RD (2017). Added value of early literacy screening in preschool children. Clin Pediatr.

[21] Aldridge MM, Goode ZD, Garbus LN, Sousa LL, Fioroni TL, Oropeza-Diaz YP (2015). Developing a toxic stress screening protocol and referral system in a large inner-city pediatric practice: an update from longitudinal data collection. Pediatrics.

[22] Berger-Jenkins E, Monk C, D’Onfro K, Sultanaet M, Brandt L, Ankam J (2019). Screening for both child behavior and social determinants of health in pediatric primary care. J Dev Behav Pediatr.

[23] Lima SS, Cavalcante LI, Costa EF (2016). Neuropsychomotor development screening of Brazilian children: a systematic review of the literature. Fisioter Pesqui.

[24] Glascoe FP (1999). Using parents’ concerns to detect and address developmental and behavioral problems. J Soc Pediatr Nurs.

[25] Oliveira CV, Palombo CN, Toriyama AT, Veríssimo ML, Castro MC, Fujimori E (2019). Health inequalities: child development in different social groups. Rev Esc Enferm USP.

[26] Silva AC, Engstron EM, Miranda CT (2015). Factors associated with neurodevelopment in children 6-18 months of age in public daycare centers in João Pessoa, Paraíba State, Brazil. Cad Saúde Pública.

[27] Fink K, Mélo TR, Israel VL (2019). Technologies in neuropsicomotor development in schools four to six years. Cad Bras Ter Ocup.

[28] Baldin MS, Apolônio AL, Vieira AR, Moretti CN, Tabaquim ML (2019). Neuropsicomotor and social affective development of babies with cleft lip/palate related to maternal mood. RIES.

[29] Silva DI, Mello DF, Mazza VA, Toriyama AT, Veríssimo ML (2019). Dysfunctions in the socio emotional development of infants and its related factors: an integrative review. Texto Contexto - Enferm.

[30] Moreira RS, Magalhães LC, Siqueira CM, Alves CR (2018). “Survey of Wellbeing of Young Children (SWYC)”: how does it fit for screening developmental delay in Brazilian children aged 4 to 58 months?. Res Dev Disabil.

